# Dynamical exploration of optical soliton solutions for M-fractional Paraxial wave equation

**DOI:** 10.1371/journal.pone.0299573

**Published:** 2024-02-29

**Authors:** Md. Habibul Bashar, Supta Ghosh, M. M. Rahman

**Affiliations:** 1 Department of Mathematics, Bangladesh University of Engineering and Technology, Dhaka, Bangladesh; 2 Department of Mathematics, European University of Bangladesh, Dhaka, Bangladesh; Korea University, REPUBLIC OF KOREA

## Abstract

This work explores diverse novel soliton solutions due to fractional derivative, dispersive, and nonlinearity effects for the nonlinear time M-fractional paraxial wave equation. The advanced *exp* [-*φ*(*ξ*)] expansion method integrates the nonlinear M-fractional Paraxial wave equation for achieving creative solitonic and traveling wave envelopes to reconnoiter such dynamics. As a result, trigonometric and hyperbolic solutions have been found via the proposed method. Under the conditions of the constraint, fruitful solutions are gained and verified with the use of the symbolic software Maple 18. For any chosen set of the allowed parameters 3D, 2D and density plots illustrate, this inquisition achieved kink shape, the collision of kink type and rogue wave, periodic rogue wave, some distinct singular periodic soliton waves for time M-fractional Paraxial wave equation. As certain nonlinear effects cancel out dispersion effects, optical solitons typically can travel great distances without dissipating. We have constructed reasonable soliton solutions and managed the actual meaning of the acquired solutions of action by characterizing the particular advantages of the summarized parameters by the portrayal of figures and by interpreting the physical occurrences. New precise voyaging wave configurations are obtained using symbolic computation and the previously described methodologies. However, the movement role of the waves is explored, and the modulation instability analysis is used to describe the stability of waves in a dispersive fashion of the obtained solutions, confirming that all created solutions are precise and stable.

## 1 Introduction

Nonlinear partial differential equations are the governing equations for a wide range of biological, chemical, and physical phenomena. Authors may gain a deeper understanding of the process being described thanks to the solution of nonlinear equations, and they may also learn facts that are not simply apparent from common observations. Exact solutions of nonlinear partial differential equations are crucial in physics and mathematics.

Now, nonlinearity is a powerful research field, and its strength is thought of through a swear-amplitude wave oscillation examined in abundant fields from optics and laser technology, shallow water waves, electrical and electronics, quantum physics, plasma physics, natural science, and biological phenomena. Different nonlinear phenomena occurring in the real world can be conveyed by way of NPDEs, and their real properties are thought of through solitonic solutions observed in several fields such as nonlinear science and engineering [1,2], bio-science [3], dual-power law [4], optics and laser technology [5], plasma physics [6], biological science [7], and etc.. Most of such phenomena in real life can be represented as nonlinear PDEs. To investigate various exact and explicit solutions there are many schemes established, such as bilinear Bäcklund transformation [8], transformed rational function method [9], tanh method [10], Bifurcation analysis [11], tanh-coth method [12], extended tanh–coth expansion method [13], multiple exp-function algorithm [14], the Kudryashov-expansion method [15], the extended Kudryashov method [16], Advanced *exp* (-*ϕ*(*ξ*))-Expansion Scheme [17], modified extended tanh scheme [18,19], MSE scheme [20], constraint and complexification method [21], Hirota bilinear [22–25], Darboux transformation [26], MEAEM [27], Generalized Darboux transformation [28], the extended direct algebraic method [29,30], Square operator method [31,32], the extended unified method [33], new auxiliary equation method [34,35], Sardar sub-equation method [36,37], Evans function method [38], a new adaptive numerical method [39], sine-Gordon expansion method [40], advanced generalized (*G*′/*G*)–expansion scheme [41], the double variable expansion method [42], the double $\left(\frac{G^{\prime }}{G},\frac{1}{G}\right)$ expansion method [43], exp (−*ϕ*(*η*))-expansion method [44], etc.

Nowadays, the fractional derivatives field is concerned in many engineering research fields. Recently, fractional order derivatives have been used in diverse real-life models of science and technology. Consequently, many researchers in fractional calculus have dedicated their devotion to recommending new fractional order derivatives, such as time fractional derivative [45,46], conformable space-time fractional [47], Riemann–Liouville fractional derivative[48], modified Riemann–Liouville fractional derivative [49], linear functional arguments using Chebyshev series [50], space–time fractional [51,52], Caputo derivative [53,54], etc.

In studying the parametric wave equation in Kerr medium, Baronio [55] utilized the one-dimensional scattering limit while considering group velocity dispersion and time-dependent space-time that lacked dimensions. The ray equation, also known as the paraxial wave equation, provides a simplified depiction of the complete wave equation and is utilized for modeling light propagation through a medium [56]. Within this context, we examine truncated time M-fractional derivative using the solution advanced *exp*[−*φ*(*ξ*)] expansion method [57] to explore some optical solutions of truncate time M-fractional paraxial wave equation [58]:

i∂P∂z+a12DkM,t2χ,ψP+a22∂2P∂y2+a3P2P=0. (1)

Where *a*_1_, *a*_2_ and *a*_3_ are real constants and *a*_1_ is dispersal effect, *a*_3_ is Kerr nonlinearity effect, and *a*_2_ is the diffraction effect. The M-fractional derivative is DkM,t2χ,ψP, and the longitudinal, transverse, and temporal propagation are denoted by variables *z*, *y*, and *t*, respectively.

Mainly as per we know we use very first time of advanced *exp*[−*φ*(*ξ*)] expansion method to explore truncated M-fractional paraxial wave equation. The fact that the majority of the time the solution is rejected due to its predefined condition is one of the limitations of our proposed method, which does not typically yield any multi-soliton solutions. The truncated M-fractional derivative is a widely recognized technique. The importance of truncated M-fractional derivative is that it fulfills the both properties of integer and fractional order derivatives. The effect of fractional order derivative on the obtained solutions is also explained by graphically. Including a fractional order term in the paraxial wave equation leads to the emergence of new optical solutions, making it a more appealing alternative to the conventional integer-order paraxial wave equation.

This work is assembled as follows: In section 2, The M-truncated fractional derivatives are described. In section 3, the working procedure of the advanced *exp*[−*φ*(*ξ*)] expansion method is enlightened; in section 4, we implemented the advanced expansion *exp*[−*φ*(*ξ*)] method into the M-truncated fractional paraxial wave equation. Section 5, describes the numerical simulations and graphical representations of some of the obtained results. In section 6, by utilizing modulation instability analysis we obtain the stability of the system. Finally, the paper concludes with a summary of its findings.

## 2 M-truncated fractional derivatives

Oliveira and Sousa proposed the M-truncated fractional derivative as a new variant of the M-fractional derivative [59]. By elimi6nating the limitations of conventional derivatives, the M-truncated fractional derivative offers a more versatile alternative.

**Definition:** Given a function $u:\left[0,\infty \right)\to ℜ$ and order *χ*, the M-truncated fractional derivative is defined as follows:

DkM,tχ,ψut=limϵ→0utκEψϵt−χ−utϵ,t>0,ψ>0.

Here, *E*_*ψ*_(*x*) is a truncated Mittag-Leffler function of one parameter, defined as [60], and taking values in the interval (0,1):

$$E_{\psi }\left(x\right)=\Sigma_{n=0}^{k}\frac{x^{n}}{\Gamma \left(\psi n+1\right)}.$$

**Characteristics:** Suppose that 0 < *χ* ≤ 1, and l,m→ℜ. Let *u*, *v* be functions that are *χ* differentiable at a point *t* > 0

1. DkM,tχ,ψlu+mv=lDkM,tχ,ψu+mDkM,tχ,ψv.Distributionlaw
2. DkM,tχ,ψuv=uDkM,tχ,ψv+vDkM,tχ,ψu.Multiplicationlaw
3. DkM,tχ,ψuv=vDkM,tχ,ψu−uDkM,tχ,ψvv2.Dividedlaw
4. DkM,tχ,ψtϖ=ϖtϖ−χ,ϖϵℜ.Powerlaw
5. DkM,tχ,ψc=0,cϵℜ.Constantlaw

If *u*, is differentiable at *v*

6. DkM,tχ,ψu∘v=u′vDkM,tχ,ψvt.

If *u*, is differentiable

7. DkM,tχ,ψut=t1−χΓψ+1dudt.

**Remarks.** Assuming that *u* is a *χ*–differentiable in the interval (0, *p*), where *p* > 0, then the following holds.


DkM,tχ,ψu0=limt→0+DkM,tχ,ψut.

## 3 A brief description of advanced *exp*(−*φ*(*ξ*))-expansion method

The nonlinear equation is expressed in terms of the M-truncated fractional derivative as follows.


ΗP,DκM,tχ,ψP,DκM,t2χ,ψP,DκM,t3χ,ψP,…=0. (2)

Suppose the following transformation

$$\begin{array}{c} \xi =l_{1}y+l_{2}z+\frac{\Gamma \left(D+1\right)}{\chi }\left(\omega t^{\chi }\right),\;\;\text{N}=v_{1}y+v_{2}z+\frac{\Gamma \left(D+1\right)}{\chi }\left(\tau t^{\chi }\right)+\delta ,\;\\ P\left(y,z,t\right)=Q\left(\xi \right)\;e^{i\text{N}}. \end{array}$$ (3)

The ordinary differential equation is derived from the given equation by utilizing the above transformation in Eq (2):

$$\text{H}\left(Q,\omega Q^{\prime },a_{1}Q^{\prime }+{a_{1}}^{2}Q^{\prime \prime },{a_{1}}^{3}Q^{\prime \prime \prime },\;\ldots \right)=0.$$ (4)

**Step-2.** According to the advanced *exp*(−*ϕ*(*ξ*)-expanssion method, the exact solution of Eq (4) is assumed to be

$$Q=\sum_{i=0}^{m}\Delta_{i}*e^{{\left(-\phi \left(\xi \right)\right)}^{i}}.$$ (5)

Where Δ_1_, Δ_2_, Δ_3_……, Δ_*m*_; Δ_*m*_ ≠ 0, are constants that to be evaluated later. The derivative of *ϕ*(*ξ*) satisfies the ODE in the succeeding system

$$\phi^{\prime }\left(\xi \right)=-\left(Se^{\left(-\phi \left(\xi \right)\right)}+Re^{\left(\phi \left(\xi \right)\right)}\right).$$ (6)

Where Δ_*i*_ are arbitrary constant. If we inject Eq (5) with Eq (6) into Eq (4), the polynomial of *e*^(*ϕ*(*ξ*))^ is obtained.

Finally, if we set the co-efficient of each term of the obtained polynomial then we achieve a system of equation. To get Δ_*i*_ we solve the system of equation. Now we substituted the obtained values of Δ_*i*_ and *Q* then we get the required solution.

The solution of the considering differential equation is given below:

**Case I:** Trigonometric function solution (when *S* > 0 and *R* > 0)
$$\phi \left(\xi \right)=ln\left(\sqrt{\frac{S}{R}}tan(\sqrt{SR}\left(\xi +C\right))\right).$$
  and
$$\phi \left(\xi \right)=ln\left(-\sqrt{\frac{S}{R}}cot(\sqrt{SR}\left(\xi +C\right))\right).$$
**Case II:** Hyperbolic function solution (when *R* < 0 and *S* > 0)
$$\phi \left(\xi \right)=ln\left(\sqrt{\frac{S}{-R}}tanh(\sqrt{-SR}\left(\xi +C\right))\right).$$
  and
$$\phi \left(\xi \right)=ln\left(\sqrt{\frac{S}{-R}}coth(\sqrt{-SR}\left(\xi +C\right))\right).$$
**Case III:** When *R* > 0 and *S* = 0
$$\phi \left(\xi \right)=ln\left(\frac{1}{-R\left(\xi +C\right)}\right).$$
**Case IV:** When *R* = 0 and S∈ℝ
$$\phi \left(\xi \right)=ln\left(S\left(\xi +C\right)\right).$$
  Where, *C* is assimilating constant.

## 4 Formation of optical solitons of Paraxial wave equation

The fractional part of the paraxial wave equation has a significant effect on the shape of the pulse, as illustrated by the following. *P*(*y*, *z*, *t*) = *Q*(*ξ*) *e*^*i*N^ in Eq (1) becomes

$$\xi =l_{1}y+l_{2}z+\frac{\Gamma \left(D+1\right)}{\chi }\left(\omega t^{\chi }\right),\;\;\text{N}=v_{1}y+v_{2}z+\frac{\Gamma \left(D+1\right)}{\chi }\left(\tau t^{\chi }\right)+\delta .$$ (7)

Where *l*_1_, *l*_2_, *v*_1_, *v*_2_, *τ* and *ω* defined as the frequencies of wave and wave numbers, N is a real function.

By applying the transformation given in Eqs (7) to (1) and then separating the resulting expression into its imaginary and real parts, we arrive at the following.


$$\left(a_{1}\omega^{2}+a_{2}l_{1}^{2}\right)Q^{\prime \prime }\left(\xi \right)-\left(a_{1}\tau^{2}+a_{2}v_{1}^{2}+2v_{2}\right)Q\left(\xi \right)-2a_{3}Q^{3}\left(\xi \right)=0.$$ (8)

And

$$\left(2a_{1}\tau \omega +2a_{2}l_{1}v_{1}+2l_{2}\right)Q^{\prime }\left(\xi \right)=0.$$ (9)

As *Q*′ (*ξ*) ≠ 0

$$l_{2}=-\left(a_{1}\tau \omega +a_{2}l_{1}v_{1}\right).$$ (10)

Apply the homogeneous balancing rule on Eq (8), we get *m* = 1.


$$Q\left(\text{T}\right)=\;\Delta_{0}+\Delta_{1}e^{\left(-\phi \left(\xi \right)\right)}.$$ (11)

Substituting Eq (11) with (6) into Eq (8), we obtain polynomial of *e*^(*ϕ*(*ξ*))^ and setting the coefficients of this polynomial equal to zero leads to the following.


$${\left(e^{\Phi \left(\xi \right)}\right)}^{0}=\Delta_{0}\alpha_{1}\tau^{2}-2\alpha_{3}\Delta_{0}^{3}-\Delta_{0}\alpha_{2}v_{1}^{2}-2\Delta_{0}\nu_{2}.$$

$${\left(e^{\Phi \left(\xi \right)}\right)}^{1}=2\Delta_{1}S\alpha_{1}\omega^{2}\text{R}+2\Delta_{1}S\alpha_{2}l_{1}^{2}\text{R}-\Delta_{1}\alpha_{1}\tau^{2}-6\alpha_{3}\Delta_{0}^{2}\Delta_{1}-\Delta_{1}\alpha_{2}v_{1}^{2}-2\Delta_{1}\nu_{2}.$$

$${\left(e^{\Phi \left(\xi \right)}\right)}^{2}=6\alpha_{3}\Delta_{0}\Delta_{1}^{2}.$$

$${\left(e^{\Phi \left(\xi \right)}\right)}^{3}=2\Delta_{1}S^{2}\alpha_{1}\omega^{2}+2\Delta_{1}S^{2}\alpha_{2}l_{1}^{2}-2\alpha_{3}\Delta_{1}^{3}.$$

Solving the aforementioned system of equations yields the following solution.


$$\omega =\pm \sqrt{-\frac{2S\text{R}\alpha_{2}l_{1}^{2}-\tau^{2}\alpha_{1}-\alpha_{2}v_{1}^{2}-2\nu_{2}\;}{2S\text{R}\alpha_{1}}};\Delta_{0}=0;\Delta_{1}=\pm \sqrt{-\frac{-S\tau^{2}\alpha_{1}-S\alpha_{2}v_{1}^{2}-2S\nu_{2}\;}{2\text{R}\alpha_{3}}}.$$

Case I: Trigonometric solutions (when *SR* > 0)

$$P_{1,2}≔\pm \frac{\sqrt{-\frac{2\left(-S\tau^{2}\alpha_{1}-S\alpha_{2}v_{1}^{2}-2S\nu_{2}\right)\;}{\text{R}\alpha_{3}}}}{2\sqrt{\frac{S}{\text{R}}}\text{tan}\left(\sqrt{S\text{R}}\;\;\left(\xi +C\right)\right)}*e^{i\text{N}}.$$

$$P_{3,4}≔\mp \frac{\sqrt{-\frac{2\left(-S\tau^{2}\alpha_{1}-S\alpha_{2}v_{1}^{2}-2S\nu_{2}\right)}{\text{R}\alpha_{3}}}}{2\sqrt{\frac{S}{\text{R}}}\text{cot}\left(\sqrt{S\text{R}}\;\;\left(\xi +C\right)\right)}*e^{i\text{N}}.$$

Where, $\xi =l_{1}y+l_{2}z+\frac{\Gamma \left(D+1\right)}{\chi }\left(\omega t^{\chi }\right)$ *and* $\text{N}=v_{1}y+v_{2}z+\frac{\Gamma \left(D+1\right)}{\chi }\left(\tau t^{\chi }\right)+\delta$.

Case II: Hyperbolic solutions (when *SR* < 0)

$$P_{5,6}≔\pm \frac{\sqrt{-\frac{2\left(-S\tau^{2}\alpha_{1}-S\alpha_{2}v_{1}^{2}-2S\nu_{2}\right)}{\text{R}\alpha_{3}}}}{2\sqrt{-\frac{S}{\text{R}}}\text{tanh}\left(\sqrt{-S\text{R}}\;\;\left(\xi +C\right)\right)}*e^{i\text{N}}.$$

$$P_{7,8}≔\pm \frac{\sqrt{-\frac{2\left(-S\tau^{2}\alpha_{1}-S\alpha_{2}v_{1}^{2}-2S\nu_{2}\right)\;}{\text{R}\alpha_{3}}}}{2\sqrt{-\frac{S}{\text{R}}}\text{coth}\left(\sqrt{-S\text{R}}\;\;\left(\xi +C\right)\right)}*e^{i\text{N}}.$$

Where, $\xi =l_{1}y+l_{2}z+\frac{\Gamma \left(D+1\right)}{\chi }\left(\omega t^{\chi }\right)$ *and* $\text{N}=v_{1}y+v_{2}z+\frac{\Gamma \left(D+1\right)}{\chi }\left(\tau t^{\chi }\right)+\delta$.

Case III & IV are rejected for the reason of their predefined condition.

## 5 Graphical explanations of the obtained result

In this section, we discuss the numerical form of the obtained solutions via the proposed schemes and their behaviors for special values of the parameter. The numerical solutions are explained graphically with 3D diagram, 2D diagram and density diagram. The diverse forms of waves are accomplished such as dark, bright, periodic, rogue, kink, double periodic and singular solitary wave results of this dynamical model. The singularity of a solution explains some properties of nonlinear media. In nonlinear optics, materials can exhibit a nonlinear response to high-intensity light. This means that the relationship between the electric field of the light and the polarization of the material is not linear. When the intensity of the light becomes very high, it can lead to phenomena like optical self-focusing, where the refractive index of the material depends on the intensity of the light. This can result in the formation of spatial solitons, which are localized, self-sustaining waves of light. The point where such effects become extreme or localized is a singularity in the solution.

**Fig 1:**
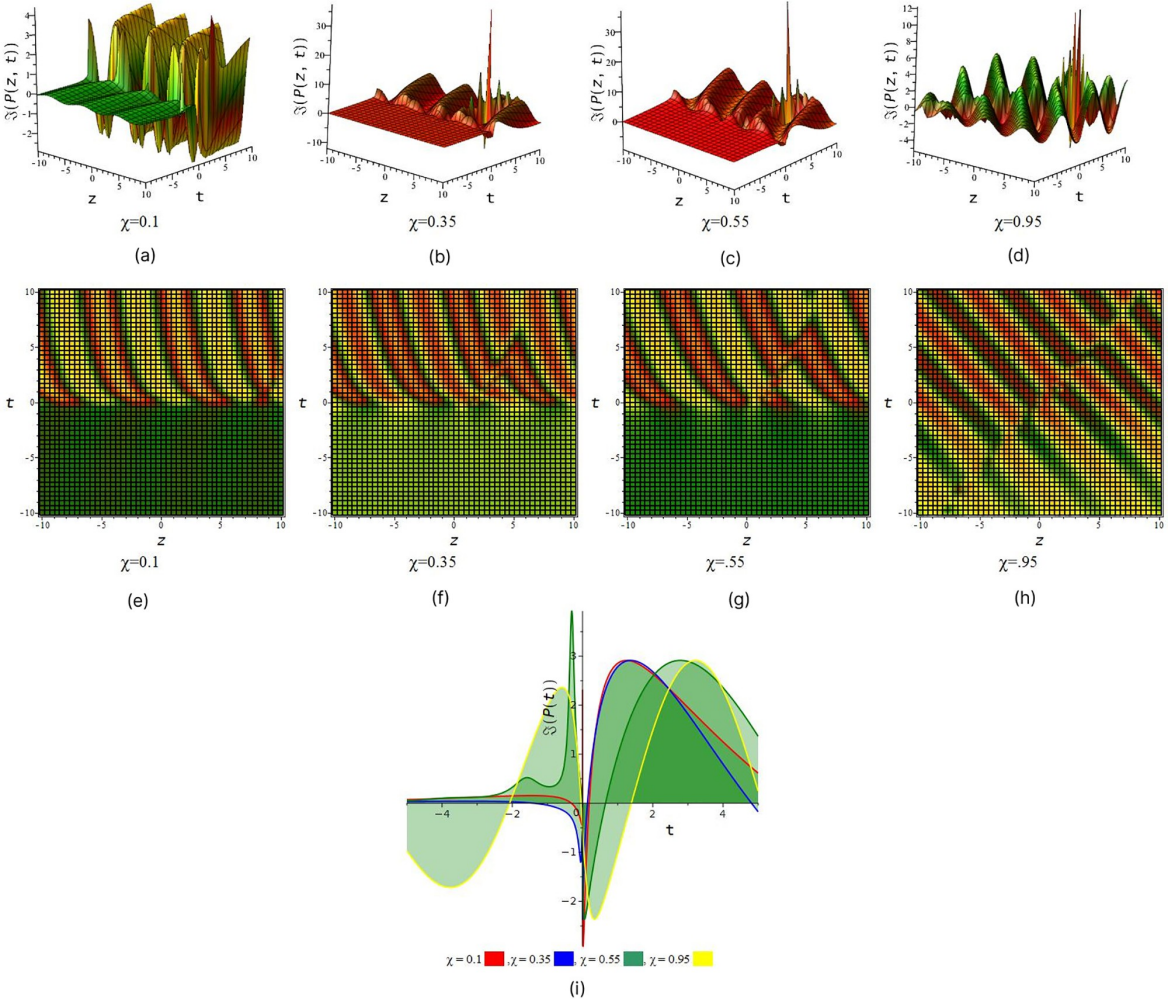
Periodic wave feature of complex part of *P*_1_. (a), (b), (c) and (d) represent three dimensional plot and (e), (f), (g) and (h) represent their corresponding density plot. And also (i) represent two dimensional plot for *z* = 0 with interval −5 ≤ *t* ≤ 5.

In Fig 1 illustrated wave structure of imaginary portion of solution *P*_1_ for suitable choice of the parametric values that *S* = 0.5, *R* = 1, *τ* = 1, *a*_1_ = 1, *a*_2_ = −5, *a*_3_ = 1, *v*_1_ = 2, *v*_2_ = 1, *l*_1_ = 1, *C* = 1, *D* = 0.6, *δ* = 1, *χ* = (01, 0.35, 0.55, 0.95) and *y* = 2 within interval −10 ≤ *z*, *t* ≤ 10.

**Fig 2:**
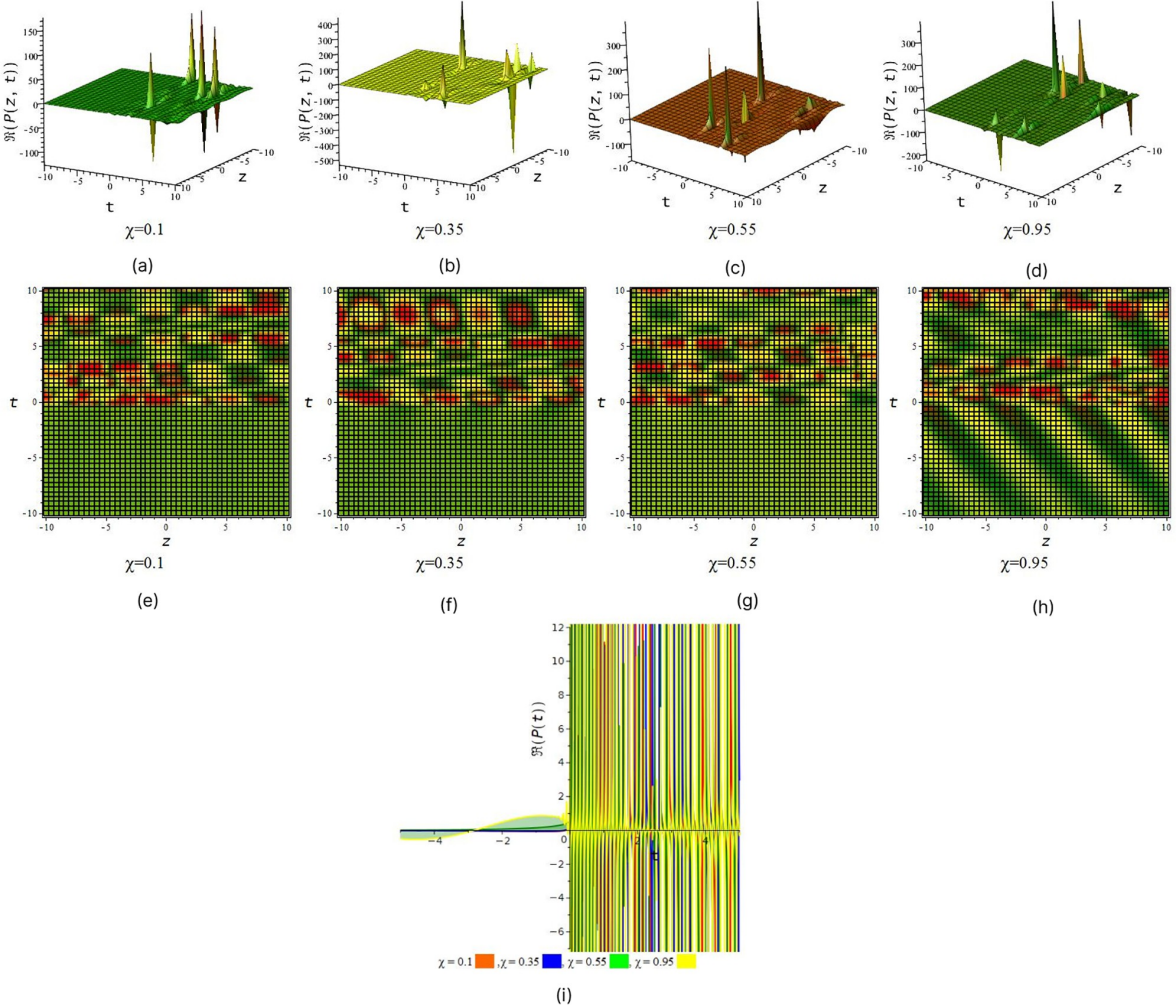
Periodic wave feature of real part of *P*_4_. (a), (b), (c) and (d) represent three dimensional plot and (e), (f), (g) and (h) represent their corresponding density plot. And also (i) represent two dimensional plot for *z* = 0 with interval −5 ≤ *t* ≤ 5.

**Fig 3:**
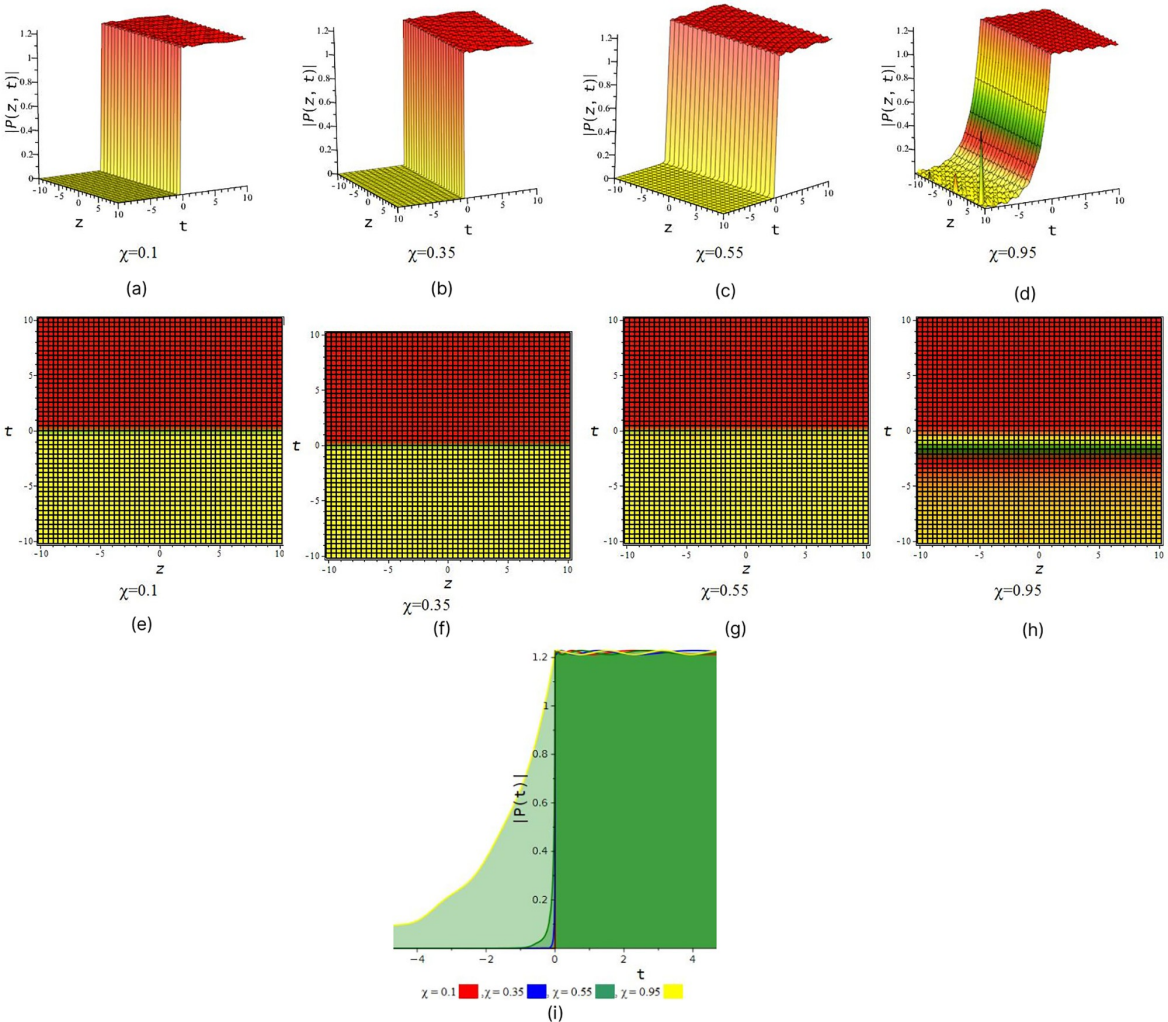
Kink wave feature of absolute part of *P*_5_. (a), (b), (c) and (d) represent three dimensional plot and (e), (f), (g) and (h) represent their corresponding density plot. And also (i) represent two dimensional plot for *z* = 0 with interval −5 ≤ *t* ≤ 5.

**Fig 4:**
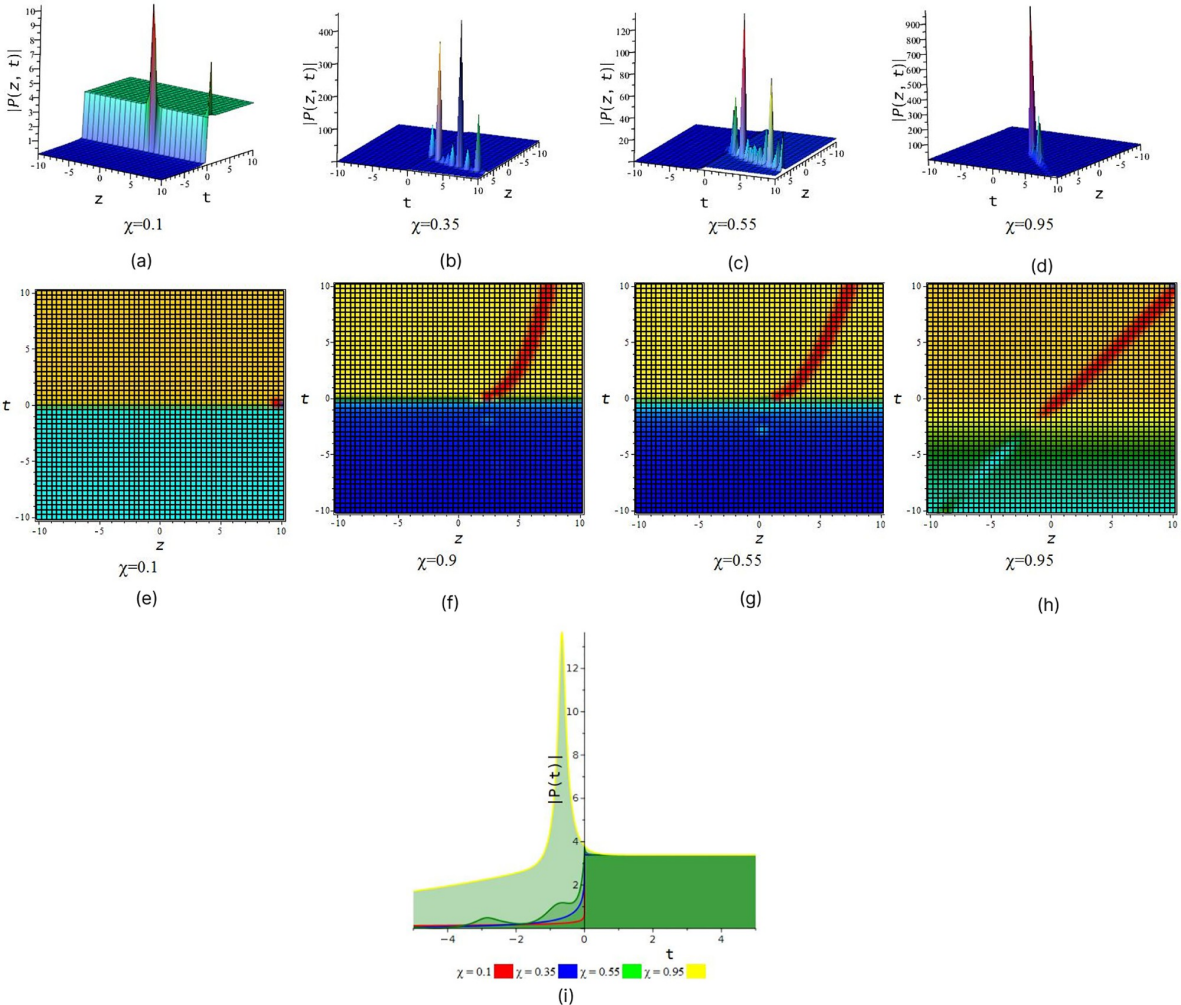
Singular kink with interaction wave feature of absolute part of *P*_5_. (a), (b), (c) and (d) represent three dimensional plot and (e), (f), (g) and (h) represent their corresponding density plot. And also (i) represent two dimensional plot for *z* = 0 with interval −5 ≤ *t* ≤ 5.

**Fig 5:**
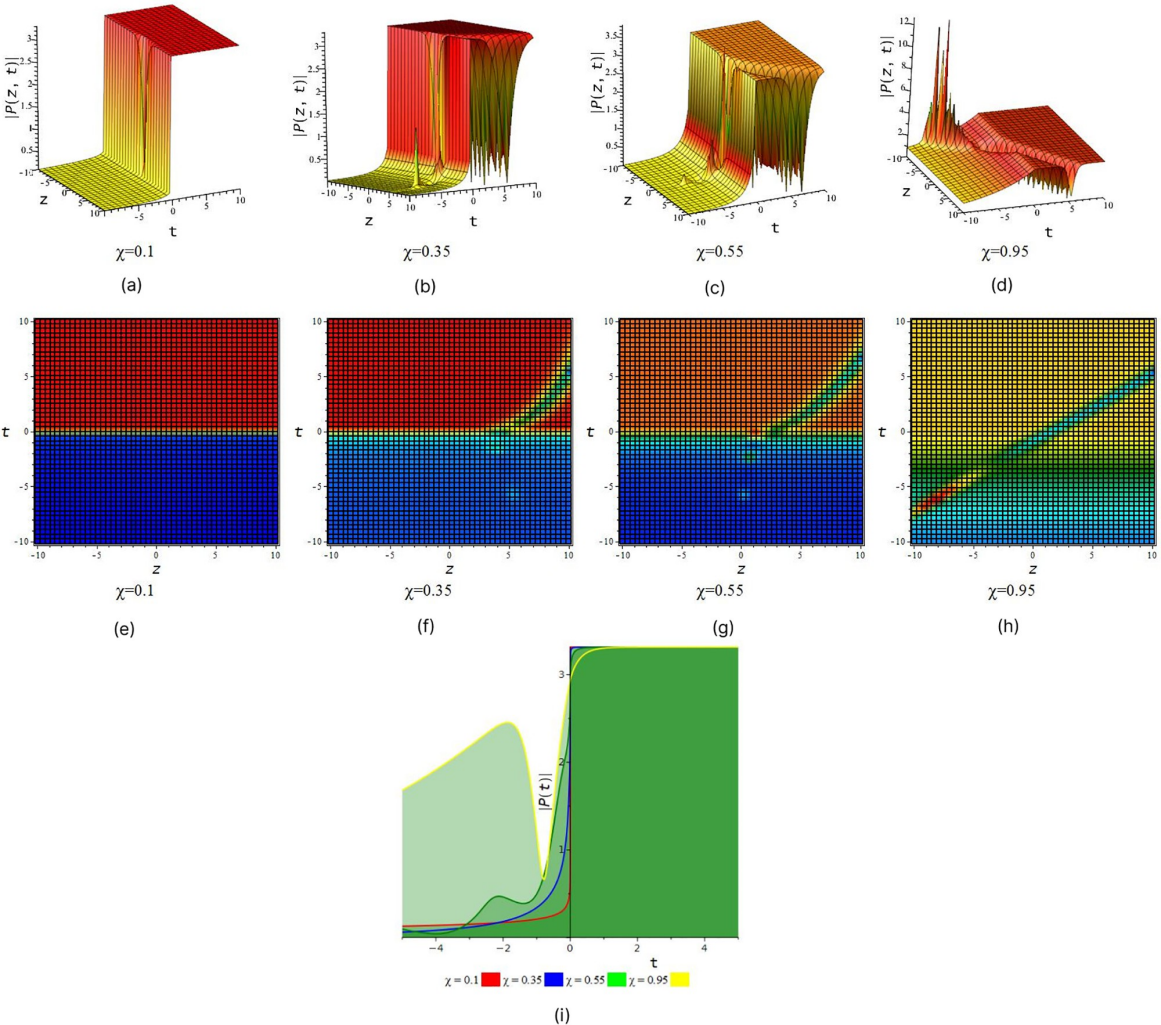
Singular kink with interaction wave feature of absolute part of *P*_7_. (a), (b), (c) and (d) represent three dimensional plot and (e), (f), (g) and (h) represent their corresponding density plot. And also (i) represent two dimensional plot for *z* = 0 with interval −5 ≤ *t* ≤ 5.

Fig 1 represent the periodic wave we also observe that after increase the value of fractional order *χ* we find out periodic wave goes interesting when *χ* = 0.35 & 0.55 rogue wave interaction occur with periodic wave and when *χ* = 0.95 the solution give look like double periodic wave. In Fig 2 illustrated wave structure of real portion of solution *P*_4_ for suitable choice of the parametric values that *S* = 0.5, *R* = 1, *τ* = 1, *a*_1_ = 0.001, *a*_2_ = −0.0005, *a*_3_ = 1, *v*_1_ = 2, *v*_2_ = 1, *l*_1_ = 1, *C* = 1, *D* = 0.6, *δ* = 0.5, *χ* = (01, 0.35, 0.55, 0.95) and *y* = 1 within interval −10 ≤ *z*, *t* ≤ 10. Fig 2 represent the periodic wave for the product of trigonometric function (cot) and exponential function we also observe that after increase the value of fractional order *χ* we find out single periodic wave goes to double periodic wave. In Fig 3 illustrated wave structure of absolute portion of solution *P*_5_ for suitable choice of the parametric values that *S* = 2, *R* = −1, *τ* = 1, *a*_1_ = −5, *a*_2_ = −0.005, *a*_3_ = −1, *v*_1_ = 2, *v*_2_ = 1, *l*_1_ = 1, *C* = 1, *D* = 2.6, *δ* = 2, *χ* = (01, 0.35, 0.55, 0.95) and *y* = 1 within interval −10 ≤ *z*, *t* ≤ 10. Fig 3 represent the kink wave for the product of hyperbolic function and exponential function we also observe that after increase the value of fractional order *χ* we find out dark kink wave goes to bright kink wave. In Fig 4 illustrated wave structure of absolute portion of solution *P*_5_ for suitable choice of the parametric values that *S* = .5, *R* = −1, *τ* = 1, *a*_1_ = −3, *a*_2_ = 6, *a*_3_ = 1, *v*_1_ = 2, *v*_2_ = 1, *l*_1_ = 1, *C* = 1, *D* = 0.6, *δ* = 0.5, *χ* = (01, 0.35, 0.55, 0.95) and *y* = 1 within interval −10 ≤ *z*, *t* ≤ 10. And In Fig 5 illustrated wave structure of absolute portion of solution *P*_7_ for suitable choice of the parametric values that *S* = 5, *R* = −1, *τ* = 1, *a*_1_ = −4, *a*_2_ = 6, *a*_3_ = 1, *v*_1_ = 2, *v*_2_ = 1, *l*_1_ = 1, *C* = 1, *D* = 0.6, *δ* = 0.5, *χ* = (01, 0.35, 0.55, 0.95) and *y* = 1 within interval −10 ≤ *z*, *t* ≤ 10. Figs 4 and 5 represent the kink wave for the product of hyperbolic function and exponential function we also observe that after increase the value of fractional order *χ* their 3D, 2D and density plot visualize that there has been acquire interaction with soliton solution.

## 6 Modulation instability analysis

A common occurrence in nonlinear partial differential equations of high order is instability, which arises from modulating the stable state due to the interplay between nonlinear and dispersive effects. In the following section, we will employ stability analysis techniques [61–63] to derive the modulation instability of the paraxial wave equation.

Let us consider the steady-state solution of the paraxial wave equation in the form:

$$P\left(y,z,t\right)=\left(G\left(y,z,t\right)+\sqrt{R}\right)e^{IRt}.$$ (12)

where *R* is the incident power. Placing Eq (12) into Eq (1) and linearizing, we get the form as below.


$$\left(I\left(\frac{\partial G}{\partial z}\right)+I\left(\frac{\partial G}{\partial t}\right)Ra_{1}-\frac{1}{2}GR^{2}a_{1}-\frac{1}{2}R^{\frac{5}{2}}a_{1}+\frac{1}{2}\left(\frac{\partial^{2}G}{\partial t^{2}}\right)a_{1}+\frac{1}{2}a_{2}\left(\frac{\partial^{2}G}{\partial y^{2}}\right)\right)+a_{3}R\left(G+\sqrt{R}\right).$$ (13)

Take the solution of the Eq (13) is as form

$$G\left(y,z,t\right)=\alpha_{1}e^{I\left(l_{1}y+l_{2}z+\omega t\right)}+\alpha_{2}e^{-I\left(l_{1}y+l_{2}z+\omega t\right)}.$$ (14)

Substituting Eq (14) into Eq (13) and collecting the coefficient of $e^{I(l_{1}y+l_{2}z+\omega t)}$ and $e^{-I(l_{1}y+l_{2}z+\omega t)}$ and by solving the determinant of the coefficient matrix, we can obtain the dispersion relation as follow:

$$l_{1}=\pm \sqrt{-R^{2}a_{1}+2R\omega a_{1}-\omega^{2}a_{1}+2Ra_{3}+2l_{2}}.$$

If −*R*^2^*a*_1_ + 2*Rωa*_1_ − *ω*^2^*a*_1_ + 2*Ra*_3_ + 2*l*_2_ ≥ 0, the value of *τ* obtained from the dispersion relation is real, then the steady state is considered stable against small perturbations. On the other hand, if −*R*^2^*a*_1_ + 2*Rωa*_1_ − *ω*^2^*a*_1_ + 2*Ra*_3_ + 2*l*_2_ < 0, *τ* turns out to be imaginary, it indicates that the perturbation grows exponentially, and the steady state becomes unstable against small perturbations. According to this condition, the modulation stability gain spectrum is obtained as:

$$H\left(spec.\right)=2Im\left(l_{1}\right)=2Im\left(\pm \sqrt{-R^{2}a_{1}+2R\omega a_{1}-\omega^{2}a_{1}+2Ra_{3}+2l_{2}}\right).$$

**Fig 6:**
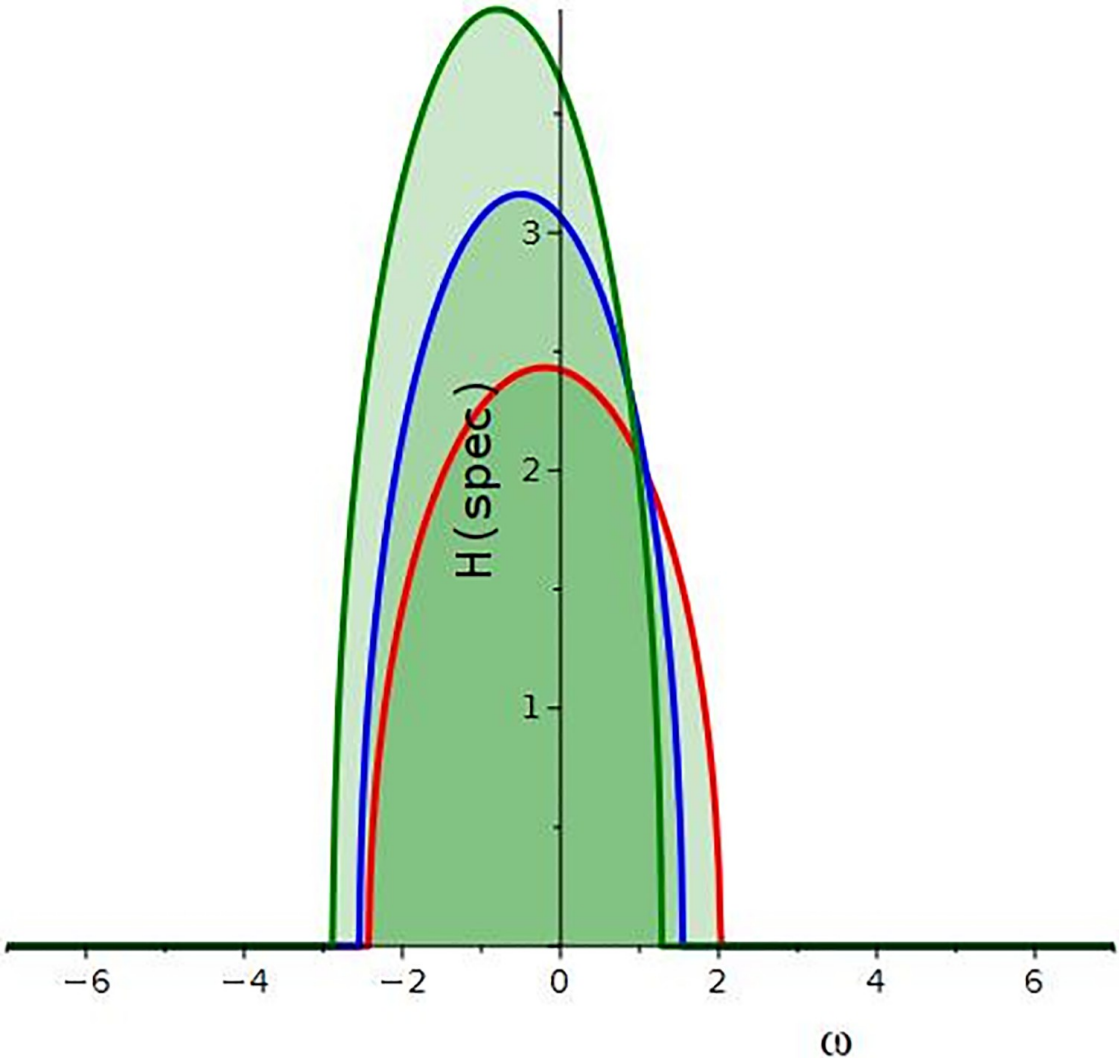
Gain spectrum of MI for different values of *a*_1_ = {−0.3, −0.6, −0.9}, *a*_3_ = {0.2, 0.5, 0.8}, *R* = {−0.2, −0.5, −0.8} and *l*_2_ = {−0.7, −1.0, −1.3}.

Fig 6 depicts the MI gain spectrum for various values of *a*_1_, *a*_3_, *R* and *l*_2_.

## 7. Conclusion

In this article, we have further developed some new exact soliton Solutions for addressing the time M-fractional paraxial wave. By executing this plan, the obtained solutions are communicated as the trigonometric and hyperbolic functions for certain free parameters. For the exceptional value of the free parameters, the obtained numerical solution provided some novel exact solutions. These solutions are illustrated in Figs 1 to 5 with three dimensional and corresponding density diagrams. We successfully shown the effect of truncated M-fractional derivative with values of the derivative parameters at *χ* = 0.1, *χ* = 0.35, *χ* = 0.55, *χ* = 0.95. This work investigates different wave design elements due to fractional derivative, dispersive, and nonlinearity effects for the nonlinear time M-fractional paraxial wave equation. By selecting various values for these parameters of the obtained solution functions, we have specifically introduced waves such as singular periodic waves, double periodic waves, kink waves and iconic solitonic waves to describe the dispersal effect, the Kerr non-linearity effect, and the diffraction effect. We used the 3D plot for better visualization, the contour plot for magnifying the direction of the wave’s velocity, and the 2D plot, which aligns the corresponding wave due to time-dependent position, to explain the nature of the wave profile of the desired solutions. It is to be noticed that these sorts of wave examination in view of the dispersal impact, the Kerr non-linearity impact and the diffraction impact may be compelling in making sense of the paraxial model related with genuine peculiarities in additional exploration. To reconnoiter such dynamics, the advanced *exp*[−*φ*(*ξ*)] expansion techniques execute to integrate the nonlinear paraxial wave model for achieving diverse solitonic and traveling wave envelops. Even though the offered method was used for the first time on the model under investigation and distinct solitons were formed, we can still achieve comparable results by selecting the same wave transformation and assigning different constant values. Therefore, the obtained outcomes expose that the projected schemes are very operative, easier and more efficient in realizing the nature of waves and such solutions of paraxial wave models are more abundant than those from other approaches. In the future, we’ll look into the non-autonomous solitons that different NLEEs might produce if their coefficients were variables also we can use spatio-temporal fractional derivation for this model.
